# Mixed Reality Technology in Total Knee Arthroplasty: An Updated Review With a Preliminary Case Report

**DOI:** 10.3389/fsurg.2022.804029

**Published:** 2022-04-15

**Authors:** Shilong Su, Pengfei Lei, Chenggong Wang, Fawei Gao, Da Zhong, Yihe Hu

**Affiliations:** ^1^Department of Orthopedics, Xiangya Hospital, Central South University, Changsha, China; ^2^Department of Orthopedics, The First Hospital of Changsha, Changsha, China; ^3^Department of Orthopedics, The First Affiliated Hospital, College of Medicine, Zhejiang University, Hangzhou, China

**Keywords:** mixed reality, augmented reality, total knee arthroplasty, navigation, case report

## Abstract

**Background:**

Augmented reality and mixed reality have been used to help surgeons perform complex surgeries. With the development of technology, mixed reality (MR) technology has been used to improve the success rate of complex hip arthroplasty due to its unique advantages. At present, there are few reports on the application of MR technology in total knee arthroplasty. We presented a case of total knee arthroplasty with the help of mixed reality technology.

**Case Presentation:**

We presented a case of a 71-year-old woman who was diagnosed with bilateral knee osteoarthritis with varus deformity, especially on the right side. After admission, the right total knee arthroplasty was performed with the assistance of MR technology. Before the operation, the three-dimensional virtual model of the knee joint of the patient was reconstructed for condition analysis, operation plan formulation, and operation simulation. During the operation, the three-dimensional virtual images of the femur and tibia coincided with the real body of the patient, showing the osteotomy plane designed before the operation, which can accurately guide the completion of osteotomy and prosthesis implantation.

**Conclusions:**

As far as we know, this is the first report on total knee arthroplasty under the guidance of mixed reality technology.

## Background

Total knee arthroplasty (TKA) is a standard treatment for severe knee osteoarthritis (1). With the aging of the population, the number of TKA is increasing every year. TKA is a difficult operation in orthopedics that requires surgeons to have abundant experience in operation and solid knowledge of anatomy. During the operation, the operator needs to consider the lower limb force line (2), soft tissue balance (3), and other aspects to ensure a good surgical effect, which is a challenge for some young surgeons or inexperienced surgeons. Moreover, for complex cases of knee osteoarthritis complicated with varus or valgus deformity of the knee joint, relying solely on the operator's experience often leads to complications such as long operation time, large surgical injury, and unsatisfactory recovery of the line of force. As a result, good post-operative results cannot be obtained (4, 5).

With the continuous progress of technology, a series of mechanical guides such as Three-dimensional (3D) printing guides are used in the intraoperative navigation of TKA. However, the mechanical guides cannot meet the requirements of accuracy in practical application, and sometimes the operation is more tedious (6). Computer navigation can improve the force line of the lower limbs and meet the requirements of accuracy, but the high cost is a big problem that hinders its wide application (7). During the operation, the surgeon needs to transfer the eyes from the operation area to the computer screen, which requires tedious technical training to complete (8). In recent years, the emergence of artificial reality technology has been a good choice to solve the above problems. Augmented reality (AR) and mixed reality (MR) technologies have been applied in the clinic and have shown great advantages. MR technology is considered to be a combination of the advantages of virtual reality technology and augmented reality technology (9). It can overlay 3D virtual images generated by the computer on the user's real-world view without the need for additional equipment to be operated during the operation. By directly covering information *in situ*, surgeons maintain the ability to interact with the real world without looking away from patients and realize accurate surgery according to the real-time guidance of 3D virtual images.

At present, MR technology has been used in liver surgery (10), vertebral compression fracture surgery (11), and total hip arthroplasty (12), and has achieved good intraoperative assistance and good post-operative results. However, in the field of total knee arthroplasty, there was no report of application. We have performed a total knee arthroplasty using MR technology. To the best of our knowledge, this was the first MR-assisted total knee arthroplasty.

## Case Presentation

**Figure 1:**
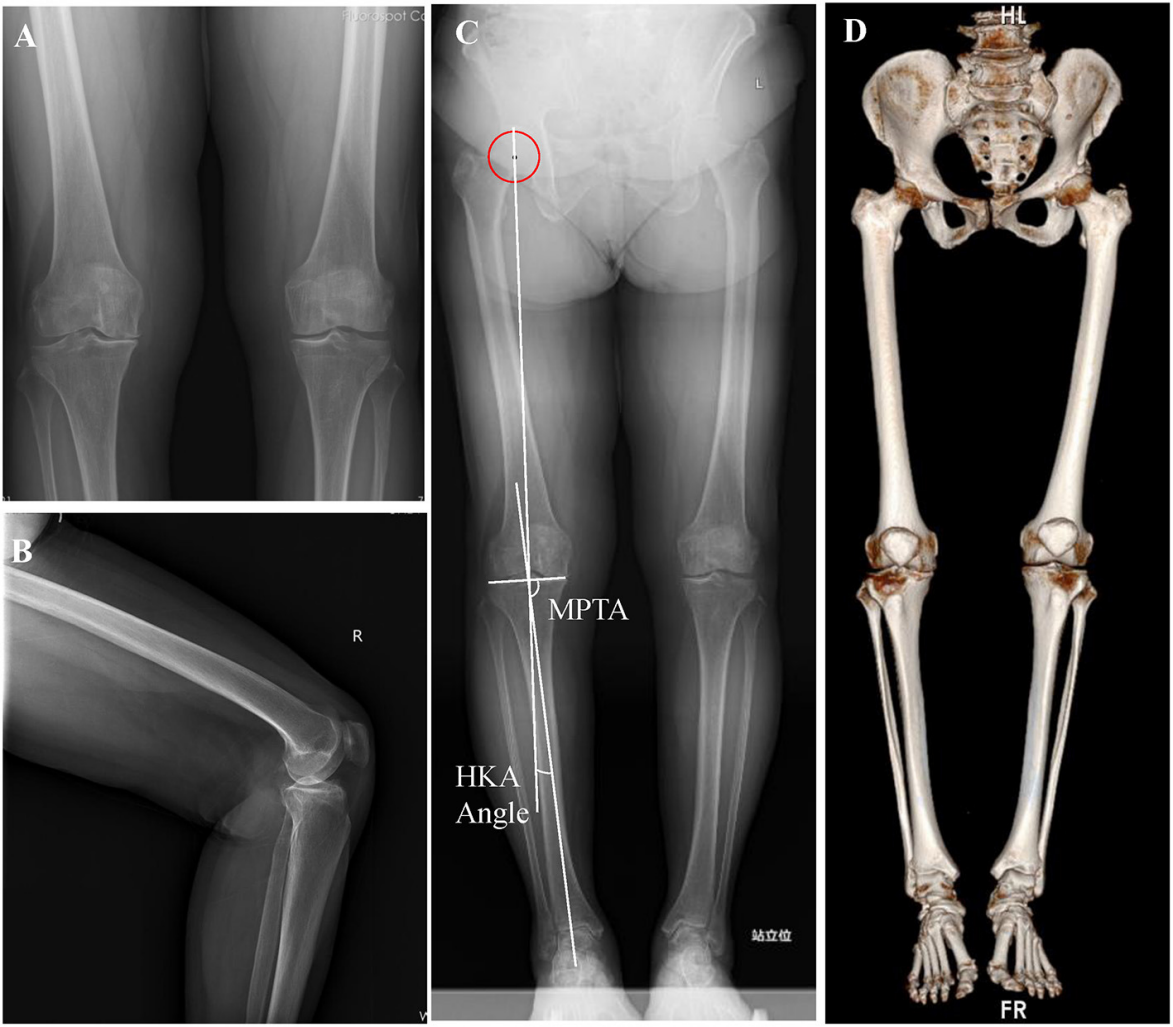
Pre-operative imaging data. **(A)** Anterior film of bilateral knee joints. **(B)** Lateral film of the right knee joint. **(C)** Full-length film of both lower limbs in heavy position. **(D)** CT 3D reconstruction of both lower extremities and pelvis.

**Figure 2:**
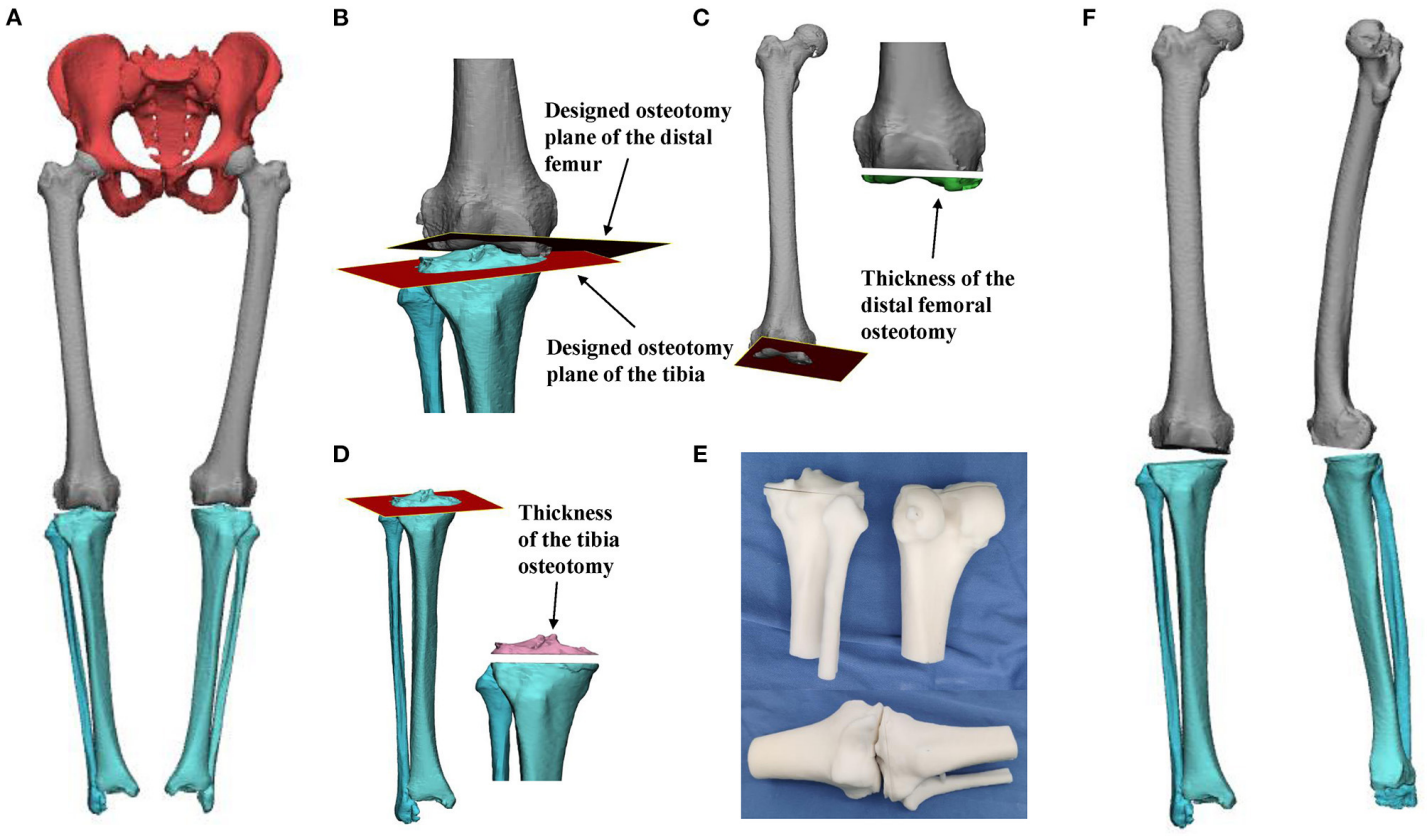
**(A)** The pelvis and both lower limbs pre-operative three-dimensional digital model, the red part is the pelvis, the gray part is the bilateral femur, the light blue part is the bilateral tibia and fibula. **(B)** The distal femur and tibia osteotomy planes are displayed. The brown part is the distal femur osteotomy plane, and the red part is the tibial osteotomy plane. **(C)** The osteotomy plane and thickness of the distal femur (cyan part). The thickness of medial femoral osteotomy was 10 mm, and the thickness of lateral femoral osteotomy was 8.3 mm. **(D)** The tibial osteotomy plane and osteotomy thickness (pink part). The thickness of medial tibial osteotomy was 3.8 mm, and that of the lateral tibia was 7.2 mm. **(E)** The 3D printing models of the distal femur and the proximal tibia and fibula were printed before the operation. **(F)** After the completion of the osteotomy, the full length of the right lower limb was viewed on the anterior and medial side.

A 71-year-old woman was admitted to hospital on May 21, 2020, because of “repeated knee pain for 10 years, aggravation on the right side with activity restriction for 5 months.” The patient had a 10-year history of the disease and received conservative treatment for a long time. The right knee joint pain was significantly aggravated within the last 5 months, accompanied by claudication and limited functional activity to seek surgical treatment. Plain X-ray and computed tomography (CT) scans showing bilateral knee osteoarthritis with varus deformity, especially on the right side (Figure 1), right hip, knee, and ankle (HKA) angle, 4 degrees, medial proximal tibial angle (MPTA), 84.8 degrees. Physical examination showed that the bilateral knee varus deformity, right flexion contracture of 20 degrees, bilateral knee joint medial tenderness, percussion pain, right knee joint range of motion of 20 to 90 degrees, left the range of motion of 0 to 120 degrees. The bilateral knee joint lateral stress test and the patellar grinding test were both positive, while the drawer test was negative. The Knee Society Score (KSS) score was 64 on the left knee and 20 on the right. Based on the medical history and physical examination results, and considering the patient's age, we decided to perform the right total knee arthroplasty. Three-dimensional virtual images before operation show severe articular cartilage wear and severe fixed flexion deformity of the right knee joint (Figure 2A), and this will make osteotomy lose important anatomical landmarks and seriously affect the accuracy of the osteotomy, especially for beginners. In the process of knee arthroplasty, there are strict requirements on the cutting angle, direction, and thickness of osteotomy, because these indicators are extremely important for the recovery of the line of force, the correct placement, and the stability of the prosthesis. Given this situation, we decided to use mixed reality technology for intraoperative guidance to ensure the accuracy of the osteotomy, in order to provide reference experience for the application of MR technology in TKA.

Before the operation, high-resolution CT (Philips, Eindhoven, Netherlands) and magnetic resonance imaging (MRI) (Siemens, Berlin, Germany) plain scans were performed to collect 3D structure data of the operation area. The CT and MRI data of the operation area (DICOM format) were imported into the computer and reconstructed by Mimics19.0 (Materialize, Leuven, Belgium) software. Different colors are used to distinguish different anatomical structures; thus, obtaining a “three-dimensional digital model of the knee joint” (Figure 2A). Using the digital model, the patient's disease characteristics are analyzed, and the operation plan is made, including the angle and direction of the distal femoral osteotomy plane (Figures 2B,C), the determination of the osteotomy plane of the tibia, and the location and size of the prosthesis (Figure 2D). Taking the medial space of the knee joint as the reference point, the thickness of medial femoral osteotomy was 10 mm, the thickness of lateral femoral osteotomy was 8.3 mm, the thickness of medial tibial osteotomy was 3.8 mm, and that of the lateral tibia was 7.2 mm, the HKA angle was 180 degrees, the distal femoral lateral angle (LDFA) was 90 degrees, the femoral condylar shaft angle (sagittal plane) was 90 degrees, the MPTA angle was 90 degrees, and the posterior tibial slope angle was 3 degrees. The digital model is printed into a solid model by using 3D printing technology (Figure 2E), as the reference instrument during the operation. The digital 3D data of the anatomy of the surgical area and the reference landmarks designed before the operation are input and opened using the helmet display HoloLens 2 (Microsoft Corporation, Redmond, W A, USA) (Figure 2F), through a personal computer (Lenovo Group, Beijing, China), and a pre-designed special automatic matching system the MiDIVI intelligent cloud platform (Jinse Medical Information Technology Co., Ltd., Changzhou, China), and the whole operation process is completely simulated to have an accurate grasp of the surgical process.

**Figure 3:**
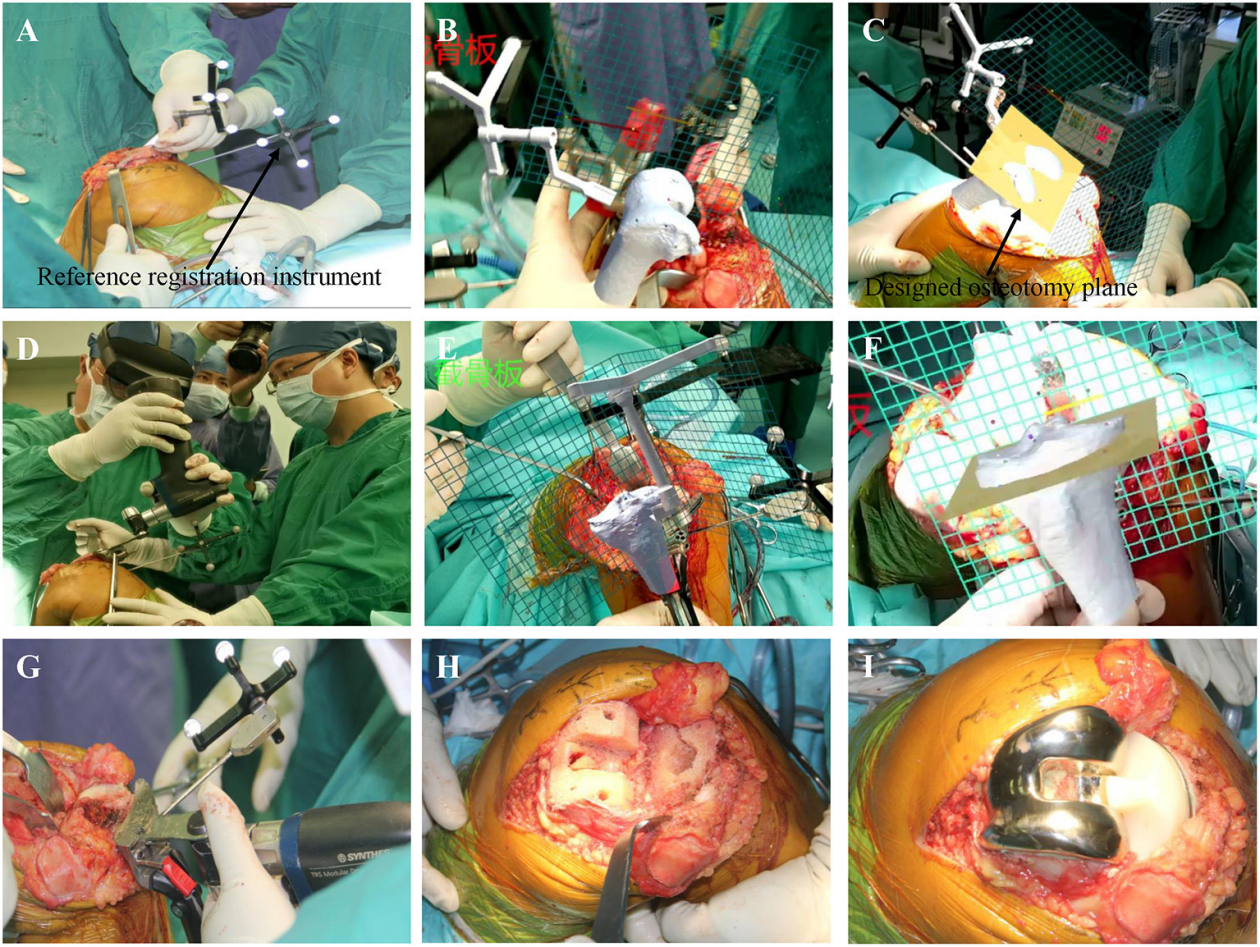
The surgical procedure and intraoperative findings: **(a)** installing a registration reference instrument on the distal femur under the guidance of the engineer. **(B)** The three-dimensional virtual proximal femur structure is coincident with the patient's real femur. **(C,D)** Under the guidance of the virtual osteotomy surface, the osteotomy of the distal femur is completed. **(E)** The three-dimensional virtual proximal tibia structure is coincident with the patient's real tibia. **(F,G)** Under the guidance of the virtual osteotomy surface, the tibia osteotomy is completed. **(H)** Osteotomy is complete. **(I)** The prosthesis has been installed.

The operation was performed using the median knee approach and the medial parapatellar approach. After lumbar plexus and sciatic nerve block anesthesia, the patient was placed in the supine position, disinfected, then the sheets were spread. When the flexion of the right knee joint is 45°, the incision through the median approach is ~15 cm, the subcutaneous tissue and muscle are separated in turn, and then the medial parapatellar incision is made to cut open the joint capsule and turn the patella to the outside. The registration method of MR technology is the volume amplification method, which has been introduced in our previous article (12) and implemented by MiDIVI intelligent cloud platform. Briefly, On a relatively constant position of the bone, we select a bony landmark and design a reference registration instrument, which is fixed with the selected bony landmark to form a whole. The registration landmarks on the reference registration instrument are identified *in vitro* to achieve registration *in vivo*, thereby enabling registration of the entire surgical field. Under the guidance of the engineer, the reference instrument for registration (Figure 3A) is installed on the side of the femur, and then, after wearing HoloLens 2, the operator opens the automatic registration software MiDIVI intelligent cloud platform for registration to coincident the three-dimensional virtual femur with the patient's real femur (Figure 3B). According to the position and direction of the osteotomy designed before the operation, the osteotomy positioning plate (Figure 3C) is installed, and the osteotomy is performed under guidance (Figure 3D). After the completion of the femoral osteotomy, the registration reference instrument is installed on the tibial side, and the three-dimensional virtual tibia is coincident with the real tibia (Figure 3E). Similarly, according to the position and direction of the osteotomy designed before the operation, the tibial osteotomy positioning plate (Figure 3F) is installed, and the osteotomy is performed under guidance (Figure 3G). The test mold was installed according to the size of the prosthesis designed before the operation, and the knee extension test showed that the knee extension space was appropriate, and the force line was satisfactory. The operative field is washed repeatedly with normal saline (Figure 3H). The tibial plateau prosthesis No. 2 is placed with bone cement (PS, Attune, DePuy Synthes, USA), followed by femoral prosthesis No. 4 until the bone cement hardened, and then 6 mm polyethylene gasket (Figure 3I) is placed. Re-examination showed that the movement of the knee joint was good, the track of the patella was good, and the line of force was satisfactory. The total operation time was 1 h and 30 min, and the intraoperative bleeding was 50 ml.

**Figure 4:**
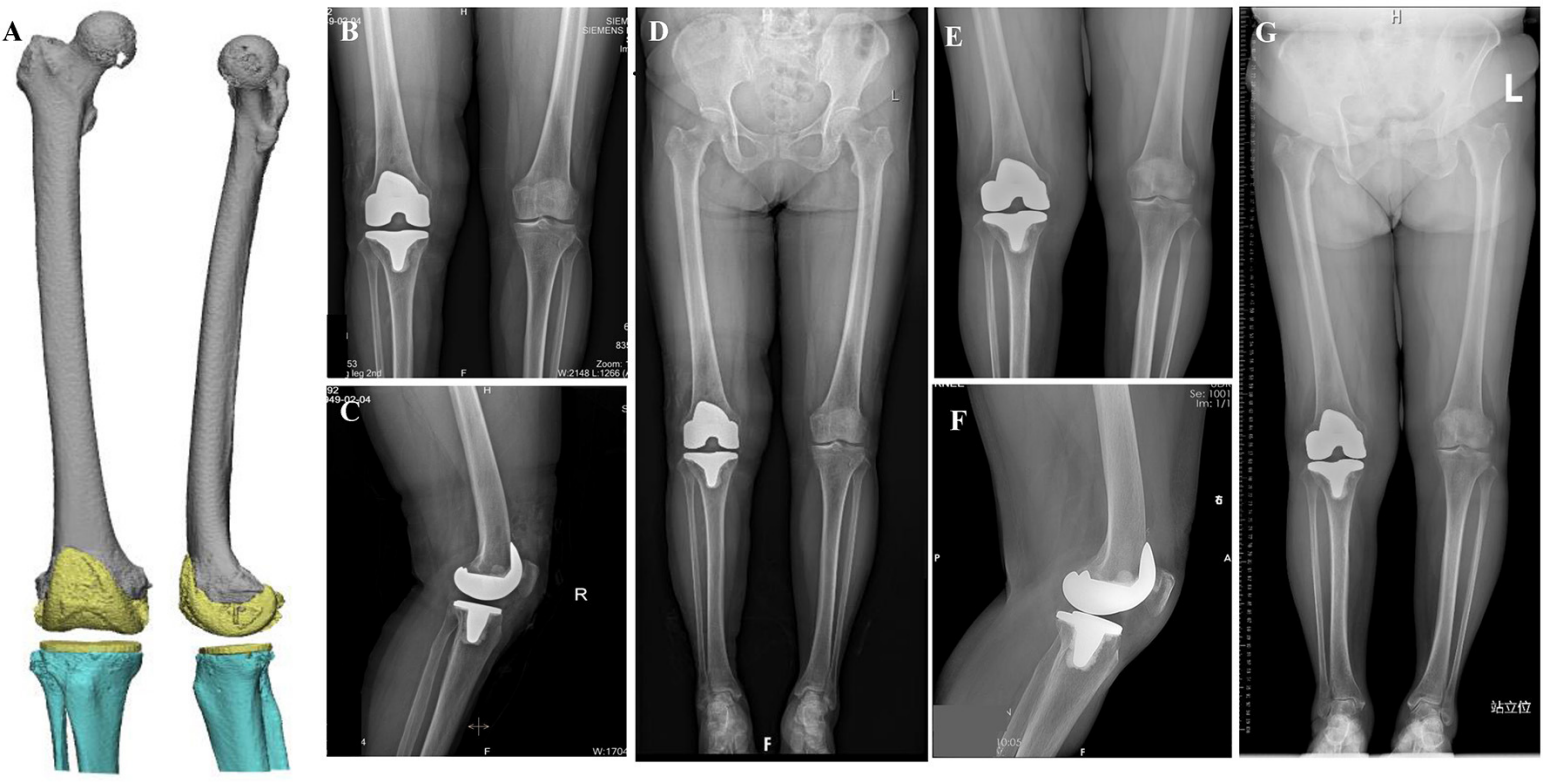
**(A)** In the anterior view and medial view of the post-operative three-dimensional digital model, the yellow part is the prosthesis. X-ray film on the second day after operation: **(B)** Anterior film, **(C)** Lateral film, **(D)** Full-length anterior position of both lower limbs. Three months after operation: **(E)** Anterior film, **(F)** Lateral film, **(G)** Full-length anterior position of both lower limbs.

Post-operatively, the patient was treated with cefoxitin to prevent infection. The post-operative CT data on the second day after the operation are imported into the computer and reconstructed using Mimics19.0 software to obtain the “post-operative three-dimensional digital model” (Figure 4A), and compared with the pre-operative operation plan. After the operation, the medial femoral osteotomy thickness of 9.6 mm, lateral of 7.3 mm, the medial tibial osteotomy thickness of 4.0 mm, lateral of 7.8 mm, the HKA angle of 180 degrees, the LDFA of 90 degrees, the femoral condylar shaft angle of 89 degrees, the MPTA angle of 90 degrees, and the posterior tibial slope angle of 5 degrees. The X-ray film on the second day after operation shows that the position of the prosthesis is satisfactory, and the force line is good (Figures 4B–D). The range of motion of the knee joint on the operative side is basically in the normal range. The second day after the operation, the patient began to walk with the help of a walker, and the recovery was good. The patient was discharged on the third day after the operation. The X-ray film 3 months after operation showed that the position of the prosthesis and the force line also was good (Figures 4E–G). And there were no obvious operative complications.

## Discussion and Conclusions

With the increasing complexity of orthopedic surgery, there are more and more tools for pre-operative planning and intraoperative guidance. The purpose of these tools is usually to improve accuracy and reduce radiation exposure to intraoperative fluoroscopy. However, the accuracy currently provided by these tools is not satisfactory; and traditional imaging and navigation systems usually display information on two-dimensional screens. Furthermore, additional equipment needs to be placed outside the surgical field. These disturbances may divert the attention of surgeons, which in turn affect the performance of surgeons (8). In recent years, a large number of advanced technologies, such as 3D modeling, 3D printing, virtual reality (VR), AR, and MR have developed rapidly in the field of surgery, which not only affect the thinking, operation, and habits of surgeons but also provide technical support for personalized precision surgery. At present, the application of AR technology in the field of orthopedics is increasing and shows great potential. At present, the field of joint surgery is mainly focused on intraoperative navigation of joint replacement. Two extracorporeal hip arthroplasty trials based on AR technology have confirmed that AR technology improves the accuracy of acetabular prosthesis placement and takes less time (13, 14). Hiroyuki Ogawa et al. designed an AR-HIP system and performed 56 hip replacements with intraoperative navigation. The results have shown that it can provide more accurate intraoperative acetabular placement angles than traditional techniques (15). Sachiyuki Tsukada (8), and Suraj Pokhrel (16) designed two tibial osteotomy navigation systems based on AR technology and preliminarily confirmed the accuracy of AR- knee navigation systems in tibial osteotomy in total knee arthroplasty. These studies have further verified the application value of AR technology in the field of joint replacement, which can provide more accurate osteotomy guidance and prosthesis implantation navigation. However, at present, the main body of technology application is AR technology, while the application of MR technology, which is the combination of VR and AR technology, is still rare, especially in knee arthroplasty. MR technology can provide more deep integration of virtual environment and real environment, as well as interaction with users, which has greater clinical application value than AR technology.

MR technology is a new type of digital holographic imaging technology that combines the advantages of VR and AR technology. By introducing real scene information into the virtual environment, it sets up an interactive feedback information loop between the virtual world, the real world, and users, to enhance the reality of user experience. Its key is to interact with the real world and obtain information promptly and to interact seamlessly with users of the real world and virtual models (9). In clinical application, it can directly reconstruct and obtain the three-dimensional structure of patients' lesions, realize the leap from a two-dimensional view to a three-dimensional view, and get rid of the limitations of a two-dimensional view. Before the operation, the operation plan can be designed and simulated based on the three-dimensional virtual image, such as how to safely cut the bone, how to design the direction and angle of the osteotomy, how to place the prosthesis, how to deal with the focus as much as possible while protecting the normal tissue structure. Most of the problems that may be encountered during the operation may be rehearsed in advance, and careful pre-operative planning can be obtained to make the operation more accurate and safe. It can realize a personalized, customized operation plan (17); besides, it can also help patients understand the operation process and promote doctor-patient communication (18). Intraoperative application is the most significant advantage of MR technology, which can superimpose the three-dimensional virtual anatomical structure image of the patient's focus area on the user's real body to achieve coincidence, to have a “perspective eye,” understand the characteristics of the lesion in real-time, and perform accurate real-time surgery according to the guidance landmarks designed before operation (11, 15, 19, 20). In general, surgical exposure is usually small, and the surgeon's gestures and accurate observation of the patient's anatomy are limited to the surgeon and his assistant. MR technology can project the surgeon's field of vision onto the screen or share the head-mounted display (HMD), that more people can see, have the opportunity to interact with colleagues, ask them for advice and guidance, and even allow remote experts to interact with surgeons in real time to achieve remote consultation (19).

At present, hologram technology, represented by MR technology, has been applied in many fields of medicine, including medical education, training of surgical skills, pre-operative planning, accurate guidance during operation, and treatment of mental illness. Roghayeh Barmaki et al. designed an anatomy learning system based on MR technology, and proved that the system is superior to traditional textbooks intervention in knowledge retention, task time, drawing results, and participation, indicating that the use of this system as a unique and powerful learning tool to supplement the standard, musculoskeletal anatomy courses, will be promising in the future (21). Sara Condino et al. discussed whether MR technology is helpful in the training of pre-operative surgical skills. Through the MR pre-operative simulation training system, the efficiency and accuracy of surgeons are improved (17). Haiyang Yu et al. made a pre-operative plan for percutaneous endoscopic discectomy by using MR technology and carried out surgical training, which greatly reduced the times of puncture and fluoroscopy, and provided a standardized method for the training of percutaneous endoscopic discectomy (22). Guan Li et al. have used MR technology to make an operation plan and assist navigation during operation to effectively improve the success rate of laparoscopic nephrectomy and reduce the interval of operation, warm ischemia time, and less estimated blood loss (20). In the field of mental illness, through the biofeedback and real-time modification of objects in the virtual environment, according to the interaction between patients, we can carry out behavioral rehabilitation based on MR technology for patients with various types of phobias and elderly people with mobility disorders living alone (23, 24).

We applied MR technology in pre-operative planning and intraoperative navigation guidance for total knee arthroplasty. The three-dimensional virtual model of the knee joint of the patient was reconstructed before the operation, the condition of the patient was analyzed stereoscopically, and the operation plan was worked out. At the same time, the operation was simulated before the operation, and the possible difficulties were predicted comprehensively, and the solution was worked out. Let patients wear HoloLens2 to communicate with the surgeon before the operation to learn about their illness and operation plan. During the operation, the three-dimensional virtual images of the femur and tibia were coincident with the real body of the patient, without the help of other mobile phones and computer equipment, only a HoloLens2; showed the osteotomy plane designed before operation after the coincidence was completed, and the osteotomy and prosthesis implantation were well-completed by relying on the accurate guidance of the virtual image in the absence of anatomical landmarks. The post-operative results were satisfactory. To evaluate the whole surgical process, MR technology-assisted total knee arthroplasty achieved an accurate individual surgery.

As an emerging technology, there are some yet unresolved problems. For example, the registration problem of MR technology is one of our pre-operative concerns. There are two traditional ways to achieve registration of MR technology. One is manual registration based on the existing structure (25), and the other is surface reconstruction registration based on an artificial reference implanted into the human body (26). However, there are problems with both methods. To resolve the problems, we have creatively combined 3D printing technology with MR technology and proposed a new solution called the “volume amplification method.” It has a good registration effect, but it needs further verification. At present, registration is still the biggest challenge for the clinical application and promotion of MR technology. In spite of this, MR technology still has a broad prospect.

As far as we know, we have completed the first case of total knee arthroplasty under the guidance of mixed reality technology, which provides a reference experience for the better application of MR technology in clinical surgery.

## Data Availability Statement

The original contributions presented in the study are included in the article/supplementary material, further inquiries can be directed to the corresponding author/s.

## Ethics Statement

Written informed consent was obtained from the individual(s) for the publication of any potentially identifiable images or data included in this article. Written informed consent was obtained from the patient for publication of this case report and any accompanying images.

## Author Contributions

DZ and PL conceived the original ideas of this manuscript. SS, CW, and FG executed the follow-up examination and materials collection. DZ, YH, and PL read the examination results and participated in the surgical and medical treatment. SS prepared the figures. PL and SS prepared the manuscript. All authors have read and approved the manuscript.

## Funding

This study was supported by National Natural Sciences Foundation of China (Grant No. 81974360 and 81902308) and The Innovation Foundation of National Orthopedics and Sports Rehabilitation Clinical Medicine Research Center (Grant No. 2021-NCRC-CXJJ-PY-37). Funding bodies (81974360, 81902308, and 2021-NCRC-CXJJ-PY-37) support the collection, analysis, and interpretation of data in this study, and the writing of this manuscript.

## Conflict of Interest

The authors declare that the research was conducted in the absence of any commercial or financial relationships that could be construed as a potential conflict of interest.

## Publisher's Note

All claims expressed in this article are solely those of the authors and do not necessarily represent those of their affiliated organizations, or those of the publisher, the editors and the reviewers. Any product that may be evaluated in this article, or claim that may be made by its manufacturer, is not guaranteed or endorsed by the publisher.

## Abbreviations

MR: Mixed reality
TKA: Total knee arthroplasty
3D: Three-dimensional
AR: Augmented reality
CT: Computed tomography
HKA, Hip, knee: and ankle
MPTA: Medial proximal tibial angle
KSS: Knee Society Score
MRI: Magnetic resonance imaging
VR: Virtual reality
HMD: Head-mounted display.
